# *Vibrio cholerae* O139 requires neither capsule nor LPS O side chain to grow inside *Acanthamoeba castellanii*

**DOI:** 10.1099/jmm.0.004721-0

**Published:** 2009-01

**Authors:** Hadi Abd, Amir Saeed, Andrej Weintraub, Gunnar Sandström

**Affiliations:** 1 Centre for Microbiological Preparedness, Swedish Institute for Infectious Disease Control, SE-171 82 Solna, Sweden; 2 Division of Clinical Microbiology, Department of Laboratory Medicine, Karolinska Institute, Karolinska University Hospital Huddinge, SE-14186 Stockholm, Sweden

## Abstract

*Vibrio cholerae*, the causative agent of cholera, has the ability to grow and survive in the aquatic free-living amoeba *Acanthamoeba castellanii*. The aim of the present study was to examine the ability of the clinical isolate *V. cholerae* O139 MO10 to grow in *A. castellanii* and to determine the effect of the bacterial capsule and LPS O side chain on intracellular growth. Results from co-cultivation, viable counts, a gentamicin assay, electron microscopy and statistical analysis showed that the association of *V. cholerae* O139 MO10 with *A. castellanii* did not inhibit growth of the amoeba, and enhanced growth and survival of *V. cholerae* O139 MO10 occurred. The wild-type *V. cholerae* O139 MO10 and a capsule mutant or capsule/LPS double mutant grew inside *A. castellanii*. Neither the capsule nor the LPS O side chain of *V. cholerae* O139 was found to play an important role in the interaction with *A. castellanii*, disclosing the ability of *V. cholerae* to multiply and survive inside *A. castellanii*, as well as the role of *A. castellanii* as an environmental host for *V. cholerae*.

## INTRODUCTION

*Vibrio cholerae* O1 and O139 have the ability to grow and survive inside the aquatic free-living amoeba *Acanthamoeba castellanii* (Abd *et al.*, 2005, 2007). However, the involvement of macromolecules in this interaction has not been studied, and it is not known whether the capsule and LPS O side chain play a role in intracellular growth and survival of *V. cholerae* in *A. castellanii*.

*V. cholerae* O139, the causative agent of cholera, possesses a mannose-sensitive haemagglutinin fimbria (Chiavelli *et al.*, 2001), a polysaccharide capsule (Johnson *et al.*, 1994; Weintraub *et al.*, 1994) and a LPS (Knirel *et al.*, 1997). Both the capsule and LPS O side chain are considered virulence factors (Waldor *et al.*, 1994) and have been found to be important factors for colonization in the mammalian intestine (Nesper *et al.*, 2002). The LPS may function as an adhesion factor (Alam *et al.*, 1997), and the capsule of *V. cholerae* O139 enhances intestinal colonization (Waldor *et al.*, 1994). Moreover, it has been shown that the capsule of *V. cholerae* O139 tends to contribute to effective adherence to the human intestinal mucosa (Yamamoto *et al.*, 1994) and to partial resistance to phagocytosis (Albert *et al.*, 1999). It is known that the mannose-sensitive haemagglutinin fimbria of *V. cholerae* O139 contributes to its attachment to plankton in aquatic habitats (Chiavelli *et al.*, 2001).

Lock *et al.* (1987) found that bacteria possessing mannose-sensitive fimbriae were able to adhere to acanthamoebae or leukocytes. Both *V. cholerae* O139 and *A. castellanii* possess ligands and receptors for mannose (Dearborn & Korn, 1974; Lock *et al.*, 1987; Tarsi & Pruzzo, 1999). It is well known that *A. castellanii* takes up bacteria via pseudopodia to form food vacuoles in which phagocytosis and digestion occur within phagolysosomes (Oates & Touster, 1976).

The aim of the present study was to examine the ability of the clinical isolate *V. cholerae* O139 MO10 to grow in *A. castellanii* and to determine the role of the bacterial capsule and LPS in the intracellular growth of *V. cholerae* O139 MO10 in *A. castellanii*.

## METHODS

### Micro-organisms.

*A. castellanii* was obtained from the American Type Culture Collection (ATCC 30010).

*V. cholerae* O139 MO10 is a wild-type strain producing a capsule and a short LPS O side chain. It is a clinical isolate and emerged in 1992 causing epidemic cholera in India. The MO10-T4 strain is a spontaneous capsule mutant of MO10 (Waldor *et al.*, 1994). The Bengal-2R strain (capsule/LPS double mutant) is a negative transposon Tn*5lac* insertion mutant of Bengal-2 (Knirel *et al.*, 1997), which is a vaccine derivative of MO10 (Waldor *et al.*, 1994). Strains were obtained from the culture collection of the Laboratory Science Division, International Centre for Diarrhoeal Disease Research, Bangladesh.

### Culture media and growth conditions.

*V. cholerae* MO10 strains were grown on blood agar plates for 24 h at 37 °C. *A. castellanii* was grown at 30 °C to a final concentration of 10^6^ cells ml^−1^ in ATCC medium no. 712. To infect the amoebae, *V. cholerae* was grown in Luria–Bertani broth to an OD_600_ of 0.6. Co-cultures of each bacterial strain and *A. castellanii* were incubated in 75 cm^2^ cell culture flasks (Corning Costar) filled with 50 ml ATCC medium no. 712 containing an initial concentration of 10^5^ cells *A. castellanii* ml^−1^ and 10^6^ cells each bacterial strain ml^−1^. Control flasks containing bacteria or amoebae only were prepared in the same way and with the same initial concentration as the co-culture flasks. The flasks were incubated without shaking at 30 °C. Samples were withdrawn regularly for microscopy, cell counts and viable counts.

The sensitivity of *V. cholerae* MO10 strains to gentamicin was determined by resuspending one bacterial colony in 1 ml sterile PBS, following a 100-fold dilution in PBS. A cotton swab was soaked in the suspension and plated on blood agar plates. One gentamicin disc (30 μg; Sigma) was applied to the agar plate, and the plate was incubated for 24 h at 37 °C. Judgement of the gentamicin sensitivity of bacteria was carried out by measuring the diameter of inhibition of bacterial growth around the gentamicin disc. A diameter ≥21 mm was considered as the cut-off point for sensitivity.

### *V. cholerae* strain association assays.

To estimate the growth and survival of the *V. cholerae* MO10 strains in the presence or absence of *A. castellanii* by viable counts, 1 ml from each bacterial control flask and from flasks containing both bacteria and amoebae was withdrawn. Viable counts were assessed by preparing 10-fold dilutions from 10^−1^ to 10^−10^ and spreading these on blood agar plates. All plates were incubated at 37 °C for 24 h.

### Bacterial adherence assay.

Co-cultures of *V. cholerae* MO10 strains with *A. castellanii* were incubated in 75 cm^2^ cell culture flasks filled with 50 ml ATCC medium no. 712 containing 100 mM mannose, an initial concentration of 10^5^ cells *A. castellanii* ml^−1^ and 10^6^ cells of each *V. cholerae* strain ml^−1^. Mannose-negative control flasks were prepared in the same way and with the same initial concentrations of co-cultivated micro-organisms but without mannose. The flasks were incubated without shaking at 30 °C and samples were withdrawn after 1 h to determine the percentage of bacteria adhered to the amoeba cells by dividing the number of amoebae with adhered bacteria by the total number of amoebae with and without adhered bacteria, multiplied by 100.

### Bacterial uptake, intracellular growth and survival.

The ability of *A. castellanii* to take up *V. cholerae* MO10 strains and the effect of the capsule and LPS O side chain on the uptake and intracellular growth of the bacterial strains were examined by comparing the interactions of wild-type MO10 and the capsule mutant and capsule/LPS double mutant of the MO10 strain with the amoebae.

Co-cultures of each bacterial strain with *A. castellanii* were incubated in 75 cm^2^ cell culture flasks filled with 50 ml ATCC medium no. 712 containing an initial concentration of 10^5^ cells *A. castellanii* ml^−1^ and 10^6^ cells each bacterial strain ml^−1^. The flasks were incubated without shaking at 30 °C for 2 h. Each cell suspension was centrifuged for 10 min at 300 ***g*** in a Labofuge GL centrifuge (VWR International) and washed six times in PBS to remove non-adhered extracellular *V. cholerae*. The pellets were resuspended in 1 ml PBS and incubated with 500 μg gentamicin ml^−1^ for 1 h at room temperature. The samples were then diluted in 9 ml PBS and centrifuged for 10 min at 300 ***g***. Each pellet was resuspended in a volume of 50 ml in a culture flask filled with ATCC medium to analyse uptake, intracellular growth and survival of *V. cholerae* strains. One millilitre from each flask was centrifuged for 10 min at 300 ***g*** and each pellet was diluted twofold with 0.1 % sodium deoxycholate to permeabilize the amoeba cells. A series of 10-fold dilutions of the sample from 10^−1^ to 10^−4^ was prepared and spread on blood agar plates. All plates were incubated at 37 °C for 24 h, and viable counts were performed for the engulfed bacteria. The reculture flasks were incubated without shaking at 30 °C to investigate the intracellular growth and survival of *V. cholerae* strains using a gentamicin assay and by viable counts for 14 days.

### Effect of mannose on the uptake of *V. cholerae* O139 MO10 in *A. castellanii*.

The uptake of *V. cholerae* MO10 by *A. castellanii* in ATCC medium containing mannose was compared with uptake in a mannose-depleted medium to determine whether bacteria adhered to the amoebae specifically via mannose or non-specifically. Both interacting micro-organisms possess ligands and receptors for mannose; thus the addition of mannose to the culture medium should inhibit specific mannose-dependent adherence between the two micro-organisms, resulting in decreased uptake and hence growth of *V. cholerae* MO10 inside *A. castellanii*.

### *A. castellanii* association assays.

Viable acanthamoebae in the presence or absence of bacteria were counted in a Bürker chamber (Merck Eurolab) under a light microscope (Carl Zeiss) using basic erythrosin B staining (ATCC). The intracellular localization of each *V. cholerae* O139 strain was analysed by electron microscopy. Five millilitre samples from culture flasks containing amoebae in the presence of bacteria were centrifuged for 10 min at 300 ***g***. The resulting pellets were washed with PBS. Each pellet of infected amoebae was fixed in 2.5 % glutaraldehyde in 0.1 M sodium cacodylate buffer (pH 7.3), with 0.1 M sucrose and 3 mM CaCl_2_,for 30 min at room temperature. Samples were then washed in sodium cacodylate buffer and post-fixed in 2 % osmium tetroxide in the same buffer for 1 h. The samples were centrifuged and the pellets were dehydrated and embedded in Epoxy resin (LX-112). The embedded samples were cut into ultrathin sections, placed on grids and stained with uranyl acetate and lead citrate. Sections were examined under a transmission electron microscope (Philips 420).

### Statistical analysis.

Student's *t*-test and a *χ*^2^ test were performed for comparative statistical analysis of growth of single and co-cultivated micro-organisms, as well as of intracellular MO10 strains, to show the role of the LPS and/or capsule of *V. cholerae* O139 in interactions with *A. castellanii*.

## RESULTS

### Growth of *A. castellanii* in the presence or absence of *V. cholerae*

To study the effect of *V. cholerae* on *A. castellanii*, growth of *A. castellanii* in the presence or absence of *V. cholerae* MO10 strains was studied by means of viable amoeba cell counts. The initial concentration of the amoebae (trophozoites and cysts) in the presence or absence of *V. cholerae* MO10 strains was 2×10^5^ cells ml^−1^, and increased 10-fold in the absence of the bacteria after 14 days.

**Fig. 1. f1:**
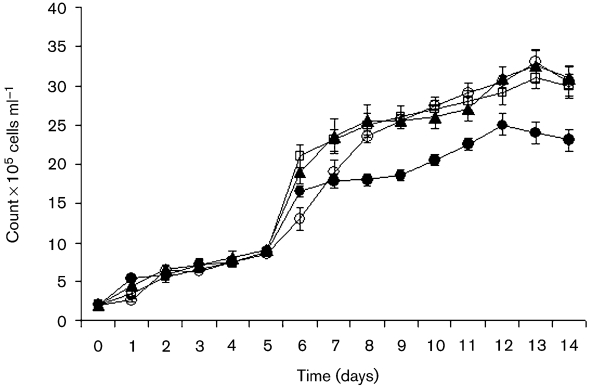
Growth of *A. castellanii*. The numbers of *A. castellanii* in the presence of *V. cholerae* MO10 (▴), *V. cholerae* MO10-T4 (•) or *V. cholerae* Bengal-2R (○), or in the absence of *V. cholerae* (□), are shown. The data indicate means±sd of four repeated experiments at each time point. The results showed that growth of *A. castellanii* is triphasic, with a log phase, a stationary phase and the start of a decline in growth.

The number of *A. castellanii* in the presence of wild-type *V. cholerae* MO10, the capsule mutant strain and the capsule/LPS double mutant of the MO10 strain also increased 10-fold (Fig. 1). Despite the number of amoebae in the presence of the capsule mutant strain, the number of amoebae was not as high as has been shown by counts in the presence of other bacterial strains. Growth of *A. castellanii* in the presence or absence of MO10 strains did not show any statistical significance (*t*-test, *P* >0.05).

### Growth of *V. cholerae* in the presence or absence of *A*. *castellanii*

To study the effect of *A. castellanii* on the growth and viability of *V. cholerae* strains, the growth and viability of *V. cholerae* strains cultivated alone in ATCC medium were compared with those of the same strains co-cultivated with amoebae in the same medium.

**Fig. 2. f2:**
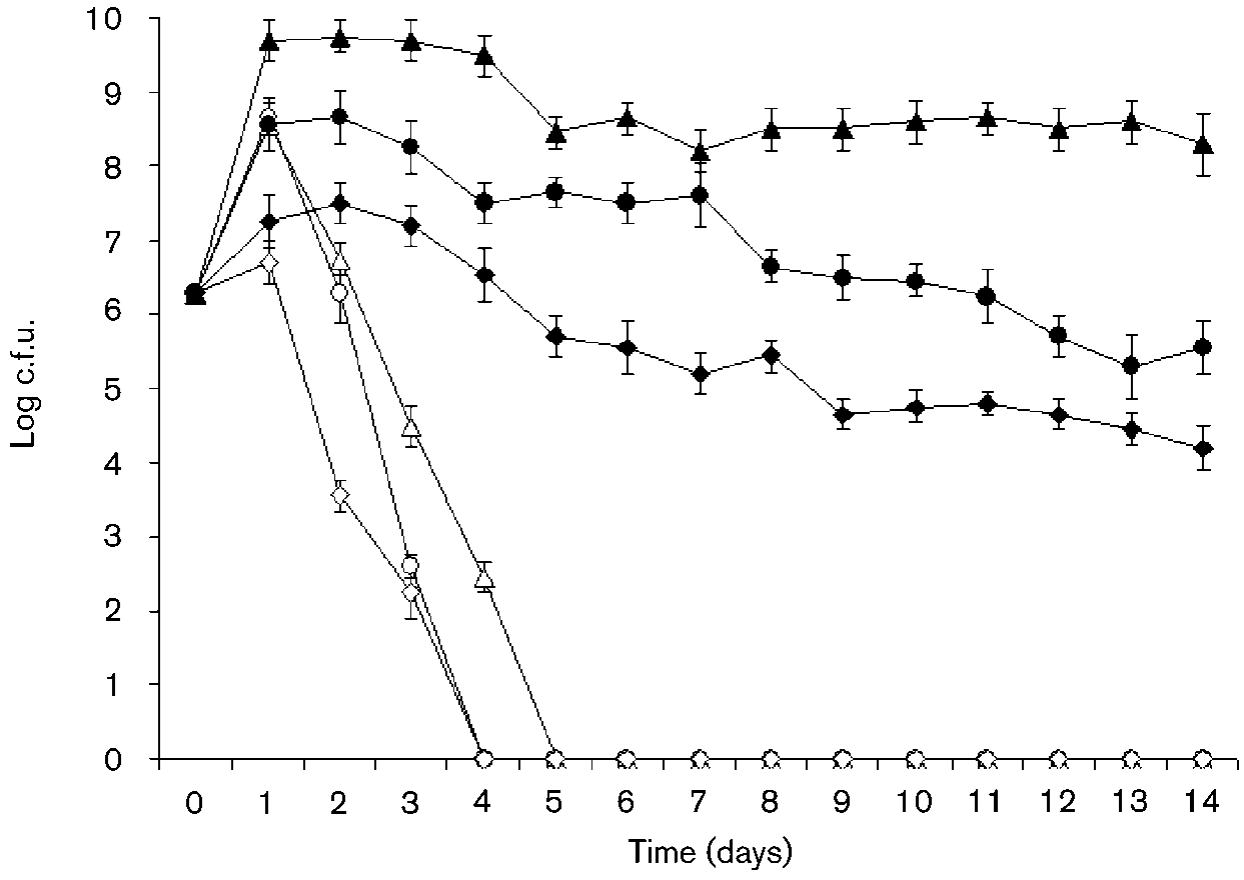
Growth of *V. cholerae*. The numbers of viable *V. cholerae* are shown in the absence (open symbols) and presence (filled symbols) of *A. castellanii* for *V. cholerae* strains MO10 (▵, ▴), MO10-T4 (○, •) and Bengal-2R (◊, ⧫). The data indicate means±sd of three repeated experiments at each time point.

The viable counts of wild-type MO10, the capsule mutant strain and the capsule/LPS double mutant strain in the presence of *A. castellanii* showed 1000-, 1000- and 10-fold increases after 1 day, respectively, and all bacterial strains survived for more than 2 weeks (Fig. 2). Viable counts of all *V. cholerae* MO10 strains in the absence of amoebae increased 100-, 100- and 10-fold during the first day, respectively, followed by a decrease to non-detectable levels on days 4 and 5 (Fig. 2). Student's *t*-test showed a significant statistical difference in the growth of bacteria in the presence or absence of *A. castellanii* (*P* <0.0001).

### Adherence between *A. castellanii* and *V. cholerae* MO10 strains

To estimate the effect of mannose on the adherence of wild-type MO10, the capsule mutant strain and the capsule/LPS double mutant strain to amoeba cells, the percentage of each bacterial strain adhering to *A. castellanii* in the presence or absence of mannose was determined and found to be 95±1.0, 98±1.5 and 93±1.0, and 96±2.0, 99±1.5 and 95±1.5 %, respectively. It was found that each amoeba cell had between one and five adhered bacteria. Moreover, adherence was not statistically significantly affected by mannose (*t*-test, *P*=0.999).

### Uptake of *V. cholerae* MO10 strains by *A. castellanii*

**Fig. 3. f3:**
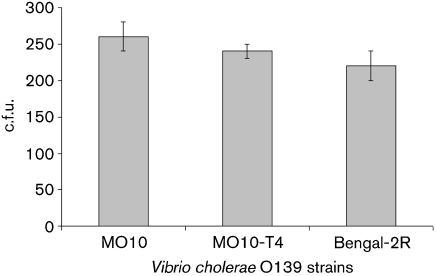
Uptake of *V. cholerae* by *A. castellanii*. The number of engulfed *V. cholerae* MO10, MO10-T4 and Bengal-2R was estimated by viable count and gentamicin assay after 2 h of co-cultivation. The data indicate means±sd of three repeated experiments at each time point.

To investigate the effect of the capsule or LPS on the uptake of bacteria, the number of engulfed wild-type MO10, capsule mutant and capsule/LPS double mutant strains was estimated by viable counts and by gentamicin assay after 2 h of co-cultivation and found to be 2.6×10^2^±20, 2.4×10^2^±10 and 2.2×10^2^±20 c.f.u. ml^−1^, respectively. The differences in the uptake of the *V. cholerae* strains were not statistically significant (*χ*^2^ test, *P*=0.99) (Fig. 3).

### Effect of mannose on the uptake of *V. cholerae* MO10

To study the effect of mannose on the uptake of the wild-type *V. cholerae* MO10 strain, uptake of MO10 by *A. castellanii* was compared in the presence and absence of mannose. The viable count of engulfed *V. cholerae* MO10 in the presence or absence of mannose after 2 h of co-cultivation was 4.0×10^2^±20 and 2.9×10^2^±10 c.f.u. ml^−1^, respectively. The uptake of *V. cholerae* MO10 in the presence or absence of mannose was not significantly different (*χ*^2^ test, *P*=0.999), thus excluding a role of mannose in the *V. cholerae*–*A. castellanii* interaction.

### Intracellular growth and survival of engulfed *V. cholerae* MO10 strains

To estimate the growth and survival of the engulfed bacteria following gentamicin treatment and recultivation, the number of bacteria growing intracellularly was estimated by viable counts.

**Fig. 4. f4:**
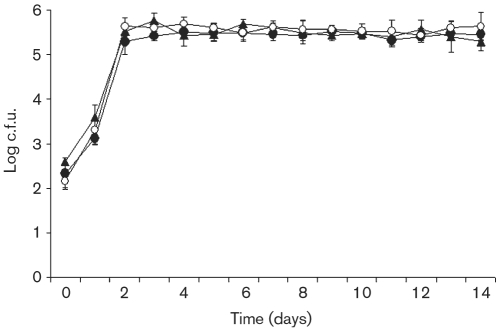
Intracellular growth and survival of *V. cholerae*. Numbers of intracellular *V. cholerae* strains MO10 (○), MO10-T4 (▴) and Bengal-2R (•). The data indicate means±sd of three repeated experiments at each time point.

Viable counts of engulfed wild-type MO10, capsule mutant and capsule/LPS double mutant strains increased intracellularly to 10^3^ c.f.u. ml^−1^ after 24 h and to 10^5^ c.f.u. ml^−1^ after 48 h, and the engulfed bacteria survived at 10^5^ c.f.u. ml^−1^ for more than 2 weeks (Fig. 4). A *χ*^2^ test did not show any statistical differences in the intracellular growth of the *V. cholerae* strains (*P*=0.999).

### Intracellular localization of *V. cholerae* MO10 strains

**Fig. 5. f5:**
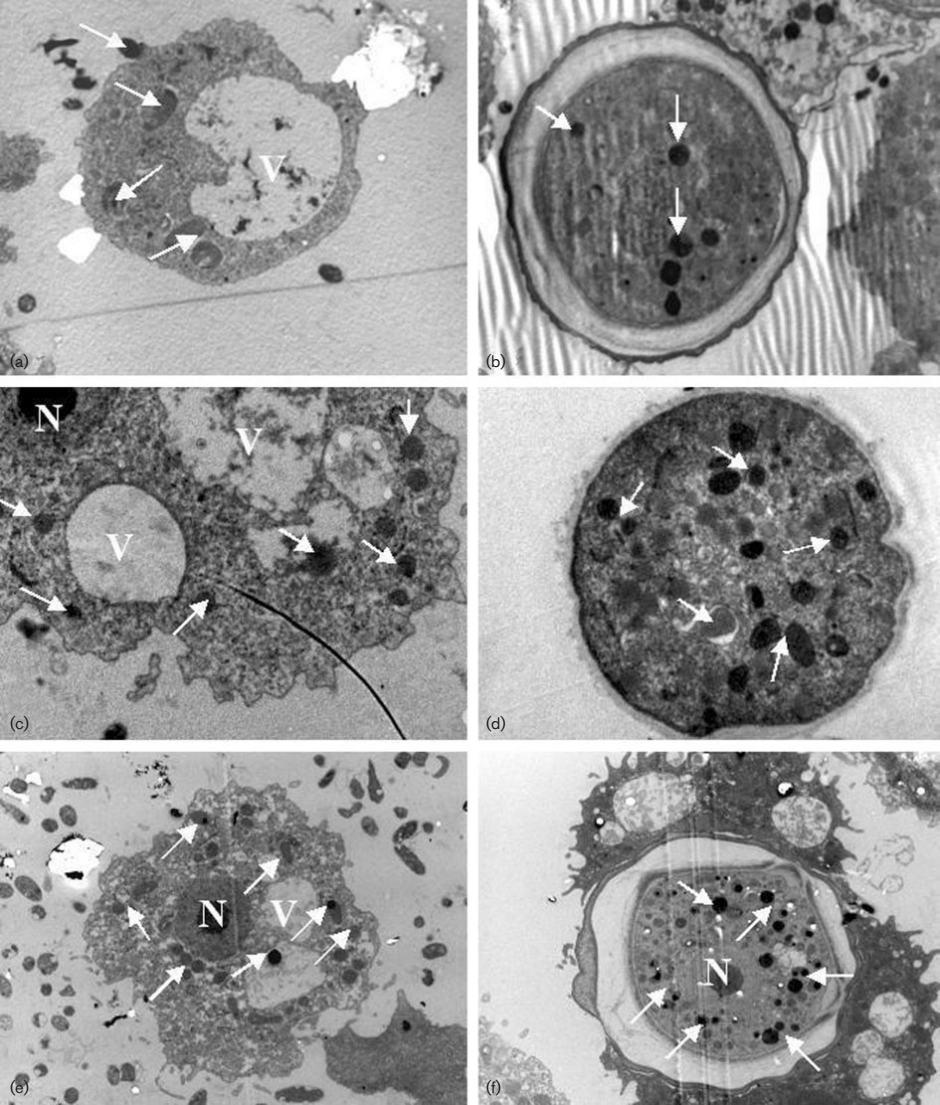
Electron photomicrographs of the intracellular localization of *V. cholerae* in *A. castellanii*. (a) *A. castellanii* trophozoite containing intracellular *V. cholerae* O139 MO10 in the cytoplasm, 3 h after co-cultivation. (b) *A. castellanii* cyst containing intracellular *V. cholerae* O139 MO10, 7 days after co-cultivation. (c) *A. castellanii* trophozoite containing intracellular *V. cholerae* MO10-T4 in the cytoplasm, 1 day after co-cultivation. (d) *A. castellanii* cyst containing *V. cholerae* MO10-T4, 6 days after co-cultivation. (e) *A. castellanii* trophozoite containing intracellular *V. cholerae* Bengal-2R in the cytoplasm, 1 day after co-cultivation. (f) *A. castellanii* cyst containing *V. cholerae* Bengal-2R, 6 days after co-cultivation. Arrows indicate bacteria. N, nucleus; V, vacuole. Magnification ×3700.

Electron microscopy was used to visualize the intracellular localization of the wild-type MO10, capsule mutant and capsule/LPS double mutant strains in *A. castellanii*. Samples from co-cultures containing *A. castellanii* and each *V. cholerae* strain were prepared separately for electron microscopy. The intracellular localization of the bacteria was in the cytoplasm of trophozoites a few hours after co-cultivation (Fig. 5a[Fig f5]). Multiplication of bacterial cells occurred in the cytoplasm of trophozoites 1 day after co-cultivation (Fig. 5c, e[Fig f5]). Moreover, bacteria were found in the cysts of *A. castellanii* 6 and 7 days after co-cultivation (Fig. 5b, d and f[Fig f5]).

## DISCUSSION

Cholera is a severe diarrhoeal disease caused by *V. cholerae* O1 or O139 (Faruque *et al.*, 2005). *V. cholerae* O1 and O139 can both grow and survive inside *A. castellanii*, indicating an endosymbiont–host interaction (Abd *et al.*, 2005, 2007; Saeed *et al.*, 2007). However, the involvement of macromolecules in this interaction has not been studied. The present study examined the ability of the clinical isolate *V. cholerae* MO10 to grow inside *A. castellanii* and determined the effect of the bacterial capsule and LPS O side chain on the intracellular growth of *V. cholerae* O139 MO10 in *A. castellanii*.

The results showed that *A. castellanii* could grow in the presence of *V. cholerae* MO10 strains and the number of amoeba increased 10-fold after 14 days of co-cultivation.

Growth of the bacteria was enhanced in the presence of amoebae compared with growth of bacteria in the absence of amoebae, which decreased to non-detectable levels by days 4–5. In this context, it was confirmed that *V. cholerae* in the absence of amoebae died during the course of the experiment and did not enter a viable but non-culturable state as has been shown previously (Abd *et al.*, 2007).

Growth of the wild-type, capsule mutant and capsule/LPS double mutant strains was enhanced in the presence of *A. castellanii* and the bacteria could grow inside the amoebae. The intracellular growth of the mutant strains was not significantly different from that of the wild-type strain.

Previous studies have shown that both the capsule and LPS O side chain of *V. cholerae* MO10 enhance adherence to human intestinal mucosa (Alam *et al.*, 1997; Yamamoto *et al.*, 1994) and that the capsule may contribute to partial resistance to phagocytosis (Albert *et al.*, 1999).

In comparison with macrophages, acanthamoebae utilize different mechanisms to capture bacteria by means of specific and non-specific adherence as well as by food-cup formation. It is well known that *A. castellanii* takes up bacteria by pseudopodia to form food vacuoles in which phagocytosis and digestion occur within phagolysosomes (Oates & Touster, 1976) or by food-cup formation and ingestion of particulate matter (Pettit *et al.*, 1996).

The adherence of bacteria to eukaryotes is the first step in the interaction between the bacterium and the host cell (Lu & Walker, 2001). Bacterial adherence to various surfaces includes several methods, including hydrophobic and ionic bonds and lectin-like interactions between bacterial ligands and complementary molecules of the substrate or receptors on eukaryotes (Lock *et al.*, 1987; Tarsi & Pruzzo, 1999). Lock *et al.* (1987) found that bacteria possessing mannose-sensitive fimbriae were able to adhere to acanthamoebae and leukocytes, and that addition of mannose completely inhibited bacterial adherence to leukocytes, whereas it only partly inhibited adherence to acanthamoebae.

It is thought that expression of fimbriae is influenced by aeration. Our cultivations were done in unshaken flasks. However, aeration also occurs in unshaken flasks, because CO_2_ is produced by live amoebae by consumption of nutrients in culture medium and disintegration of part of the amoebal cells, as has been observed in interactions between *Francisella tularensis* and *A. castellanii* (Abd *et al.*, 2003). Moreover, the culture flask had a filtered cap, which permitted air exchange.

It is known that the mannose-sensitive haemagglutinin fimbria of *V. cholerae* O139 contributes to its attachment to plankton in aquatic habitats (Chiavelli *et al.*, 2001). In this context, our result showed that the addition of mannose did not affect the adherence to *A. castellanii* or the uptake and intracellular growth of *V. cholerae* MO10 in *A. castellanii*. This supports the findings by Lock *et al.* (1987) and may indicate that specific adherence mediated by other factors such as the outer-membrane protein and the toxin co-regulated pilus, as well as non-specific adherence, participate in the interaction between *V. cholerae* and *A. castellanii*.

In spite of the fact that *V. cholerae* O1 El Tor possesses a mannose-sensitive haemagglutinin fimbria and *V. cholerae* O1 classical does not (Hanne & Finkelstein, 1982), their intracellular growth in *A. castellanii* is not significantly different (Abd *et al.*, 2007).

The results showed that the clinical isolate *V. cholerae* MO10 grew and survived symbiotically in *A. castellanii* over the course of the experiment. The intracellular growth of the wild-type, capsule mutant and capsule/LPS double mutant strains was not significantly different, despite differences in the cell composition of each strain. Moreover, mannose depletion did not inhibit the intracellular growth of wild-type *V. cholerae* MO10 in *A. castellanii*. Thus *V. cholerae* cells may adhere non-specifically to *A. castellanii* in addition to specific adherence mediated by factors other than mannose.

Acanthamoebae and pathogenic bacteria such as *V. cholerae* are present worldwide in aquatic environments (Behets *et al.*, 2007), including drinking water (Backer, 2002; Brown & Barker, 1999; Greub & Raoult, 2004), and the use of water with poor microbiological quality increases the risk of human illness (Snelling *et al.*, 2006). Acanthamoebae and bacteria are involved in complex interactions important to medical and environmental microbiology. In this context, it may be beneficial to discuss briefly the possible effects of the outcome of the interaction between amoebae and bacteria on health. It is known that acanthamoebae benefit from extracellular bacteria as food (Weekers *et al.*, 1993), which may enhance survival of the amoebae in different environments. In contrast, the role of acanthamoebae as hosts for bacteria has been proposed for many pathogenic bacteria (Abd *et al.*, 2003, 2005, 2007; Alsam *et al.*, 2006; Axelsson-Olsson *et al.*, 2005; Barker *et al.*, 1999; Cirillo *et al.*, 1994; Gaze *et al.*, 2003; Jeong *et al.*, 2007; La Scola & Raoult, 2001; Ly & Muller, 1990; Steinert *et al.*, 1998; Winiecka-Krusnell *et al.*, 2002). The acanthamoebae support bacterial growth and survival (Saeed *et al.*, 2007) and save the bacteria from the effects of chlorination (King *et al.*, 1988) and antibiotics (Abd *et al.*, 2003, 2005, 2007), increasing the risk of human illness caused by the bacteria or acanthamoebae. Accordingly, a need to find an effective means of killing the intracellular bacteria is warranted under these circumstances, to help reduce the risk of spread of *V. cholerae* and other bacteria.
